# Validations of the microchannel flow model for characterizing vascularized tissues

**DOI:** 10.3390/fluids5040228

**Published:** 2020-11-30

**Authors:** Sedigheh S. Poul, Juvenal Ormachea, Stefanie J. Hollenbach, Kevin J. Parker

**Affiliations:** 1 Department of Mechanical Engineering, University of Rochester, Rochester, NY 14627, USA; 2 Department of Electrical and Computer Engineering, University of Rochester, Rochester, NY 14627, USA; 3 Department of Obstetrics and Gynecology, University of Rochester Medical Center, Rochester, NY 14623, USA

**Keywords:** microchannel flow model, elasticity, shear wave speed, stress relaxation time constant, placenta, vasoconstriction

## Abstract

The microchannel flow model postulates that stress-strain behavior in soft tissues is influenced by the time constants of fluid-filled vessels related to Poiseuille’s law. A consequence of this framework is that changes in fluid viscosity and changes in vessel diameter (through vasoconstriction) have a measurable effect on tissue stiffness. These influences are examined through the theory of the microchannel flow model. Then, the effects of viscosity and vasoconstriction are demonstrated in gelatin phantoms and in perfused tissues, respectively. We find good agreement between theory and experiments using both a simple model made from gelatin and from living, perfused, placental tissue *ex vivo*.

## 1. Introduction

Tissue elasticity measurements can provide valuable information related to pathological changes in tissues. Therefore, elastography has been extensively applied to the diagnosis of normal and abnormal conditions of tissues such as breast, liver, prostate, thyroid and placenta [1,2]. For example, Athanasiou et al. [3], in a study on 48 women with benign or malignant breast lesions, showed that elasticity of malignant lesions was higher than benign cases. Nabavizadeh et al. [4] compared the complex elasticity parameters such as storage and loss moduli and also the loss tangent of malignant versus benign breast tumors, and stated that these parameters could serve as potential biomarkers. Wang et al. [5] reported a significant correlation between liver stiffness and fibrosis scores in 320 patients. Bavu et al. [6] in a study on 113 patients with hepatitis C virus used supersonic shear imaging (SSI) and showed that the SSI elasticity assessments are useful for evaluating fibrosis stages of the livers. In a study to investigate the tissue elasticity measurements for characterizing the normal versus cancerous prostate, Hoyt et al. [7] reported that the stiffnesses of cancerous prostate were significantly higher than the normal cases and suggested that tissue elasticity could be used as a cancer detection biomarker. Edwards et al. [8] summarized several studies that investigated the use of placental elasticity measurements in clinical applications. Kılıç et al. [9] in a study on 50 pregnant women showed that the placenta stiffness in patients with preeclampsia is substantially higher than the control groups. Hollenbach et al. [10] have shown that ischemic placental disease affects the shear wave speed (SWS), which correlates with elasticity, and suggested that elasticity imaging offers potential for placental disease discrimination.

Estimating the elasticity of tissues using appropriate mathematical/physical models is a fundamental need for reliable elastography measurements, and there have been different studies focused on that topic [11-20]. The microchannel flow model (MCFM) was proposed by Parker [21] as a descriptive and predictive model for elasticity quantification in soft, vascularized tissues. Under the assumption of the MCFM framework, the stiffness and SWS of vascularized tissues are related to the vasculature network parameters, the vessels’ fluid flow under stress, as well as the tissue parenchymal characteristics. In the derivation of the MCFM, time constants are introduced by employing Poiseuille’s flow law for the fluid inside the vessels, which is dependent on the fluid viscosity and the vessel diameter. Some pathological conditions could cause a change in the fluid flow parameters inside the vessel network, which under the MCFM framework reflects changes in the values of the time constant parameter associated with the vasculature. For example, inflammation in the liver [22], or the preeclampsia condition (a medical disorder in pregnancy that is associated with high blood pressure and the fluid flow reduction) in placenta [23,24] could both cause vessel constriction.

In this paper, we present validations of the MCFM theory focusing on the fluid vessel time constants. Specifically, the effects of change in fluid viscosity and also vessel diameter (through vasoconstriction) on the time constants and therefore, on tissue stiffness, are investigated. The results are based on the gelatin phantom experiments having fluid channels inside, and also *ex vivo* perfused placenta that is considered to be a highly vascularized tissue.

## 2. Theory

### 2.1 Background

**Figure 1.**
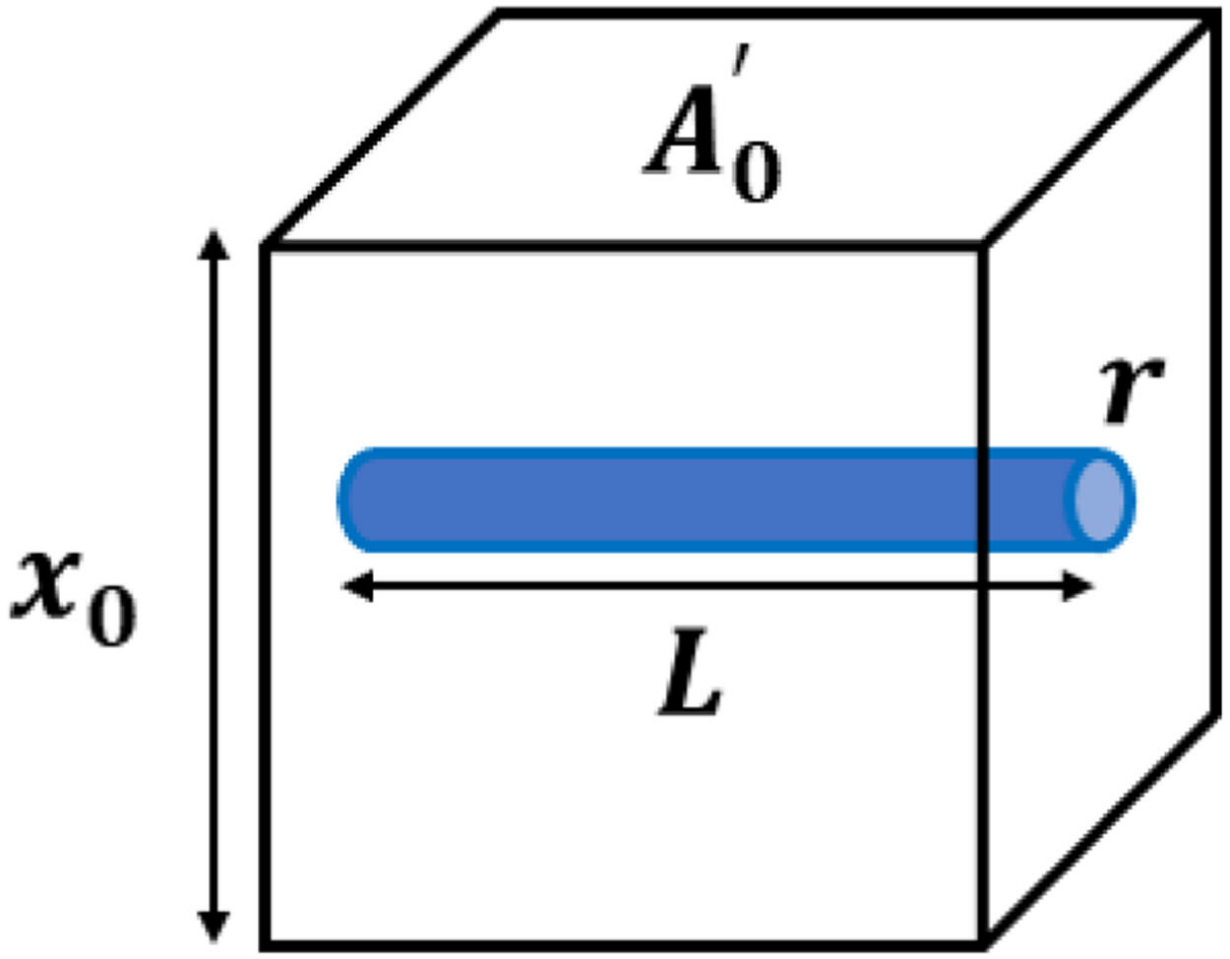
An ideal model of an elastic, isotropic block with a single interior fluid-filled channel is considered for the stress derivation in the MCFM. It is assumed that the channel is open to free exit of fluid volume only on one side, and the flow is governed by Poiseuille’s law.

The MCFM [21,25] begins with the analysis of a simple scenario, shown in Figure 1, where an elastic homogeneous material contains a small fluid-filled channel open on only one side.

When placed in a stress relaxation test, we assume the resulting strain must account for both the elastic strain and the loss of volume as fluid is squeezed out of the channel under compression. A key assumption is that the fluid flow will be governed by Poiseuille’s law for laminar flow which states that the fluid flow undergoes a pressure drop of Δ*P* according to Equation (1), which is dependent on the fluid viscosity (*η*), flow volume rate (*Q*), channel radius (*r*), and channel length (*L*). On the other hand, this pressure drop is physically proportional to the normal stress *σ_x_* from the compression test sensed at the interior side of the microchannel (Δ*P*≅*C*_1_*σ_x_*), with *C*_1_ as a constant.

(1) $$\Delta P=\frac{8\eta L}{\pi r^{4}}Q≅C_{1}\sigma_{x}$$

Assuming the decrease in volume of the fluid in the channel is reflected as the decrease in the volume (the length along the *x*-coordinate) of the tissue around it, the stress-strain relationship for the fluid-filled channel is derived in Equation (2) and simplified as Equation (3), which is analogous to a dashpot stress-strain relationship.

(2) $$\frac{d\varepsilon }{dt}=\frac{Q}{A_{0}^{\prime }x_{0}}=\frac{C\sigma_{x}r^{4}}{A_{0}^{\prime }x_{0}\eta }$$

(3) $$\sigma_{x}(t)=\eta \left(\frac{A_{o}^{\prime }x_{0}}{cr^{4}}\right)\cdot \frac{d\varepsilon }{dt}$$

In these equations, *ε* is the strain applied, $A_{{}_{0}}^{{}^{\prime }}$ is the cross-sectional area of the tissue block, *x*_0_ is the dimension along *x*, and *C* is a constant equal to *π*/8*L*. Considering the elastic medium and the fluid-filled channel as two elements undergoing relaxation results in a simple series spring-dashpot model with a single exponential decay given in Equation (4) with the characteristic time constant associated with the fluid-filled channel *τ_fluid_* as in Equation (3).

(4) $$\sigma_{x}(t)=\varepsilon_{0}Ee^{-\frac{t}{\tau_{fluid}}}$$

(5) $$\tau_{{}_{fluid}}=\left(\frac{\eta A_{o}^{\prime }x_{0}}{cr^{4}}\right)/E$$

In Equations (4) and (5), *E* is the Young’s modulus of the elastic medium, and *ε*_0_ is the magnitude of constant step strain applied. For the case of a vascularized tissue model, there will be a distribution of fluid channels with different radii corresponding to the distribution of vessels that result in having a spectrum of time constants, all contributing to the stress relaxation behavior. Using the superposition principle and integrating stress over a continuous distribution of relaxation time constants (channel sizes) assumed to follow a power law behavior *A*(*τ*) = *A*_0_*τ*^*b*^, we have:

(6) $$\sigma_{SR}(t)=A_{0}t^{1-b}\Gamma \left[b-1\right]$$

which is the two-parameter stress relaxation equation with *b* as the key power law parameter related to the fractal vasculature structure and *A*_0_ as a constant, in which Γ is the gamma function. Equation (6) is derived for a distribution of time constants from zero to infinity, however, in practice there are bounds on the largest and smallest vessels in a vasculature corresponding to larger veins and arteries and small capillaries and extracellular spaces, which map to the lowest and highest characteristic time constant, respectively. Applying these bounds on the integration over the relaxation time constant, the stress relaxation follows a four-parameter equation as shown in Equation (7) with two additional parameters of *T*_max_ and *T*_min_ where *a* = *b* − 1.

(7) $$\sigma_{{}_{SR}}(t)=A_{0}\cdot \frac{\Gamma \left(a,\frac{t}{T_{{}_{\max}}}\right)-\Gamma \left(a,\frac{t}{T_{{}_{\text{min}}}}\right)}{t^{a}}$$

This framework makes specific predictions about the behaviors when either viscosity is changed, or vasodilation or vasoconstriction causes a change in vessel radii.

### 2.2 Change in the Relaxation Time Constant Associated with the Fluid Type

In theory, when viscosity is changed in the simple channel-in-phantom (CIP) model shown in Figure 1, we simply have a change in the stress relaxation time constant and the stress level. In the derivation of the MCFM, each channel contributes to the relaxation with a distinct characteristic time constant according to Equation (5). The time constant increases linearly with the increase in the viscosity of the fluid inside the CIP and decreases with the stiffness level of the phantom; the more viscous fluid and also the softer background case have higher time constants. Therefore, a change in the fluid viscosity would have the effect of changing the stress relaxation response in experiments, assuming all other parameters remain constant.

### 2.3 Change in the Relaxation Time Constant Associated with Vessel Size

By definition, any vasoconstriction or vasodilation in vascularized tissues such as placenta impose a change in the vessel radii. Introducing a scale factor *χ*, the relationship between the radii of the changed states after vasoconstriction/vasodilation *r*_2_ and the initial state *r*_1_ is modeled as:

(8) r2=χr1{χ>1vasodilationχ<1vasocontriction}

The state of the vasculature after vasoconstriction/vasodilation have a new relaxation spectrum *A*_2_ (*τ*_2_) as well as new maximum and minimum time constants denoted by *T*_max,2_ and *T*_min,2_, respectively. The parameters of the second states of vasculature are related to that of the initial states by employing the general transformation rule explained in detail in Parker [26], which are shown in Equation (9) (which is true for *T*_max,2_ and *T*_min,2_ as well) and Equation (10).

(9) $$\tau_{2}=\frac{\tau_{1}}{\chi^{4}}$$

(10) $$A_{2}(\tau_{2})=\chi^{4}A(\tau_{1})=\chi^{4}A(\tau_{2}\chi^{4})$$

The stress relaxation response for the new vasculature is obtained by integrating over the new relaxation spectrum, shown in Equation (11).

(11) $$\sigma_{{}_{SR}}(t)=A_{0}\chi^{4(b-1)}\frac{\Gamma \left(a,\frac{t}{T_{{}_{\max,2}}}\right)-\Gamma \left(a,\frac{t}{T_{{}_{\text{min},2}}}\right)}{t^{a}}$$

The vessel radii scale factor *χ* is found as the key parameter in linking the two states of the vasculature before and after undergoing the vasoconstriction/vasodilation. Vasoconstriction, characterized as the case with *χ* < 1, causes an increase in time constants as well as a general hardening of the tissue in comparison to the baseline state.

Therefore, vasoconstriction/vasodilation experiments in living tissues undergoing changes in the vessel radii are another way to study the effect of changes in the characteristic time constants on the tissue response.

### 2.4 Ramp-plus-hold Models for CIP

**Figure 2.**
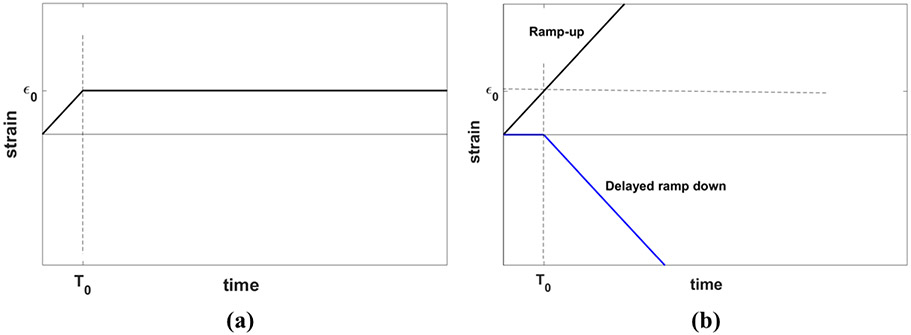
**(a)** An experimental step strain function (ramp-plus-hold), and **(b)** an equivalent representation of the strain function in **(a)** by a ramp-up plus a delayed ramp-down strain function that occurs when the two ramps are superimposed.

The stress relaxation behavior of a material is characterized by its response to the application of an ideal step strain function on the sample of the material. However, *ideal* step strain involves imposing an instantaneous change of strain from zero to the desired strain which, in practice, cannot be achieved physically. Therefore, step strain is modeled as a combination of two processes: first, a gradual linear increase of strain from zero to a desired strain level *ε*_0_ in the form of a ramp with the duration of *T*_0_, and second, keeping the strain *ε*_0_ constant, allowing the viscoelastic material to relax in time, as show in Figure 2 (a), similar to the derivation of stress response using the Kelvin-Voigt fractional derivative (KVFD) model by Zhang *et al* [27].

Applying ramp-plus-hold strain is equivalent to the superposition of ramp-up strain and delayed ramp-down strain, as depicted in Figure 2 (b). The same rule applies to the stress response according to Equation (3):

(12) $$\sigma_{{}_{ramp+hold}}(t)=\sigma_{{}_{ramp\text{-}up}}(t)+\sigma_{{}_{ramp\text{-}down}}(t-T_{{}_{0}})\cdot Heaviside(t-T_{0})$$

From systems theory, the response of a linear system to the ramp input is the integral of the response to the *ideal* step input. Thus, for the CIP with fluid, the *ideal* step response in Equation (13) is employed to derive Equation (14) as the CIP ramp response. Equation (13) expresses the stress contribution from a CIP filled with fluid in response to the *ideal* step strain with the time constant of *τ_fluid_* introduced by the CIP, and *B*_0_ is a constant.

(13) $$\sigma_{{}_{ideal\;step}}(t)=B_{0}e^{-\frac{t}{\tau_{fluid}}}$$

(14) $$\sigma_{{}_{ramp}}(t)=B_{{}_{0}}\left(\tau_{{}_{fluid}}-e^{-\left(\frac{t}{\tau_{fluid}}\right)}\tau_{{}_{fluid}}\right)$$

By substituting Equation (14) into Equation (12), the CIP ramp-plus-hold response can be easily obtained. To focus mainly on the role of the fluid inside the vessels in the characteristic time constant, we consider two experiments where the elastic block is the same for both cases, but the viscous fluid in the CIP is different. If subscripts (1) and (2) correspond to the cases with fluid (1) and fluid (2), respectively, then the stress difference *σ*_2_ (*t*)−*σ*_1_ (*t*) between these two cases when subjected to ramp-plus-hold strain is given by:

(15) $$\Delta \sigma (t)=\sigma_{2}(t)-\sigma_{1}(t)=\left\{B_{2}\left(\tau_{2}-e^{-\left(\frac{t}{\tau_{2}}\right)}\tau_{2}\right)+B_{2}\left(\tau_{2}-e^{-\left(\frac{t-T_{0}}{\tau_{2}}\right)}\tau_{2}\right)Heaviside(t-T_{0})\right\}\;\;-\left\{B_{1}\left(\tau_{1}-e^{-\left(\frac{t}{\tau_{1}}\right)}\tau_{1}\right)+B_{1}\left(\tau_{1}-e^{-\left(\frac{t-T_{0}}{\tau_{1}}\right)}\tau_{1}\right)Heaviside(t-T_{0})\right\}$$

where Δ*σ*(*t*) denotes the difference between the total stresses for the two cases.

## 3. Methods

### 3.1. Phantom Preparation

**Figure 3.**
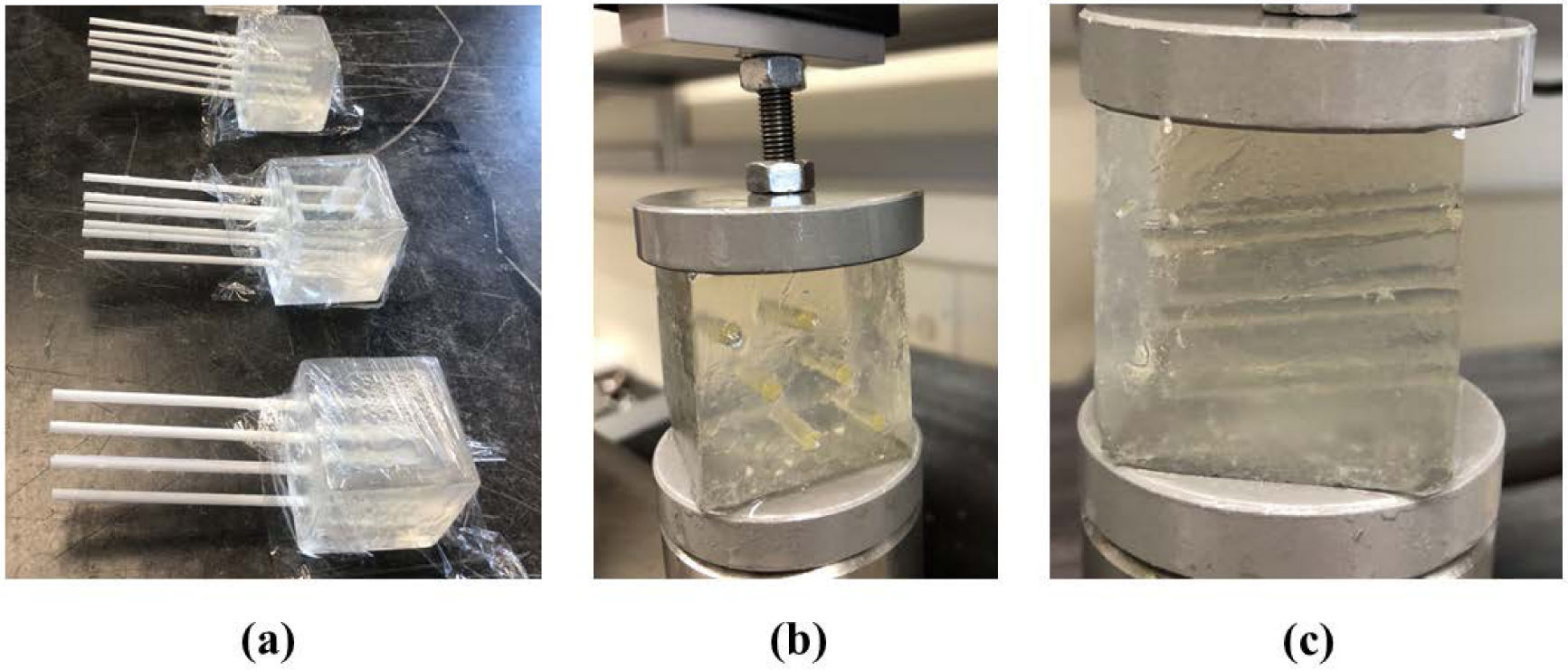
**(a)** Samples of gelatin-based phantoms each including six CIPs. **(b)** and **(c)** The same samples undergoing the stress relaxation test after injecting castor oil inside the CIPs.

For making the CIP samples, four small plastic rectangular cubes were made as the molds with a cross section of 35 mm × 35 mm and a height of approximately 38 mm. Each mold had 6 circular slots with diameters of 3 mm for inserting disposable plastic straws to make the CIPs. The arrangement of the CIPs (or straws) are shown in Figure 3 (a) and (b). The channels are placed inside the cubes at a slight angle of approximately 5° as shown in Figure 3 (c) to avoid the fluid flow out of the CIPs during the experiment.

Gelatin-based phantoms are made from a specific mixture of gelatin, agar, sodium chloride (NACL) in 900 mL of degassed water with the weight proportions of the ingredients listed in Table 1 [28].

The phantom preparation procedure was similar to the method used in [28] and is described here. For making a homogeneous gelatin phantom, the gelatin powder, agar, and NaCl were mixed and the mixture was added slowly to the degassed water while stirring to let the ingredient dissolve. Then, the mixture was heated up to a temperature of about 65° C and then was placed on a magnetic stirrer to stir it constantly. By placing an ice bath around the container, the solution was allowed to cool down to nearly 30° C. The straws were sealed using wax and then inserted in the cubic mold before adding the gelatin mixture into the molds. The samples were left at a temperature of 4° C overnight to let the gelatin mixture solidify. On the next day, the phantoms were allowed to reach room temperature before performing the mechanical testing.

### 3.2 Stress Relaxation Test

The compression test for stress relaxation measurements was done by applying a 5% strain on each sample using a Q-Test/5 machine (MTS, Eden Prairie, MN, USA) with a 5 N load cell and a compression rate of 0.15 mm/s for approximately 500 seconds of relaxation time. The ramp-plus-hold time was approximately 12-13 seconds depending on the sample height. The test was done on 3-4 samples of gelatin phantoms with castor oil and also with olive oil as the CIP fluid. The relaxation force measured during the test was converted to stress using the cross-sectional area of the samples. Then, the difference in the stress variation in time of the castor oil CIP samples and the olive oil CIP samples was fitted to a stress vs. time relationship similar to Equation (15) to obtain the coefficients. A slightly modified version of Equation (15) was used in the curve-fitting procedure with an additive constant *δ* included. This additional constant allows for some flexibility in the fitting process and accounts for small experimental sample-to-sample differences.

Stress curve fittings were performed using the curve-fitting toolbox in MATLAB (MathWorks, Inc., Natick, MA, USA) based on the nonlinear least square minimization of errors.

### 3.3 Viscosity Measurement

Noting that the viscosity of the fluid plays a major role in the value of the time constant, the viscosities of the two fluids employed are measured at lab temperature using a viscometer (Cannon, model 9721-B74, universal size 400, State College, PA, USA). Using the time period required when a fluid inside the viscometer flows between two marked points and also the characteristic viscometer constant provided by the manufacturer, the kinematic viscosity of the two fluids are calculated and then converted to the dynamic viscosity *η* for each fluid using the density of the fluids. The characteristic viscometer constant is 0.4511 cSt/s at room temperature.

### 3.4 Placental Elastography Images and SWS Measurements

Elastography images of the *in vitro* perfused human placenta were obtained at a center frequency of 5 MHz using a Siemens Antares scanner (Siemens Medical Solutions, Malvern, PA, USA) and a VF10-5 probe (Siemens Medical Solutions, Malvern, PA, USA) with a single-track location shear wave elasticity imaging (STL-SWEI) pulse sequence and accelerated processing [29]. To study the shear wave propagation in the placenta, the velocity versus time data were obtained at two locations after applying a radiation push pulse and then, by employing the Fourier transform, the amplitude and phase of the velocity were obtained as a function of frequency.

Placenta tissue under this study was from a healthy pregnancy immediately after caesarean section with no overall defects observed during post-delivery examination. Using an open perfusion system, an arterial pressure of < 40 mmHg and a normal fetal flow at about 3-6ml/min were maintained. For creating *in vitro* vasoconstriction in the perfused placenta, a dose of 1 ml 10^−6^ M vasoconstrictor agent (thromboxane agonist, U46619, Caymen Chemical Co., Ann Arbor, MI, USA) was injected in the fetal artery.

The protocol for this study was approved by the Research Subjects Review Board at the University of Rochester. The placenta is a disposable tissue specimen from the surgical examinations and no patient is identified related to the disposable samples. Therefore, consent from the patient was not needed, consistent with the World Medical Association Declaration of Helsinki.

## 4. Results

The results are presented in two sections: first, the results from the stress relaxation tests on the CIP gelatin phantoms with fluid channels are presented. The second section includes elastography results on the perfused placenta in baseline and vasoconstriction condition

### 4.1 Phantom Experiments

#### 4.1.1 Viscosity Measurements

The viscosities of castor oil and olive oil are measured using the viscometer and the average of measurements and the standard deviation for each case are reported in Table 2. The measurement for each oil is repeated 4-5 times and the low standard deviation for both cases indicates the reproducibility of the measurements. The results show that castor oil is almost 12 times more viscous than olive oil.

#### 4.1.2 Stress Relaxation

**Figure 4.**
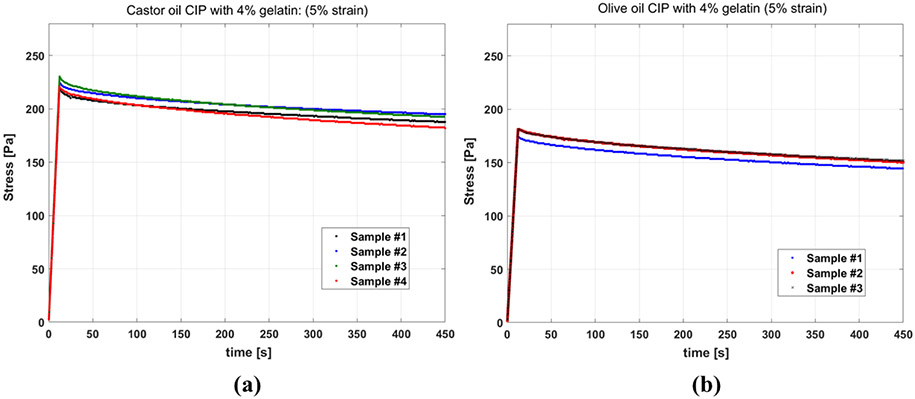
Stress relaxation curves for phantoms with: **(a)** castor oil CIPs (four samples) **(b)** olive oil CIPs (three samples).

Figures 4 (a) and (b) show the results of stress relaxation tests on gelatin-based phantoms with castor oil and olive oil channels, respectively. The stress curves of different samples of the same fluid inside CIP being similar indicates the good reproducibility of the test. Also, comparing the stress level for the two groups, it is observed that the samples with castor oil have elevated stress in comparison to the samples with olive oil, and appear stiffer in the experiment.

**Figure 5.**
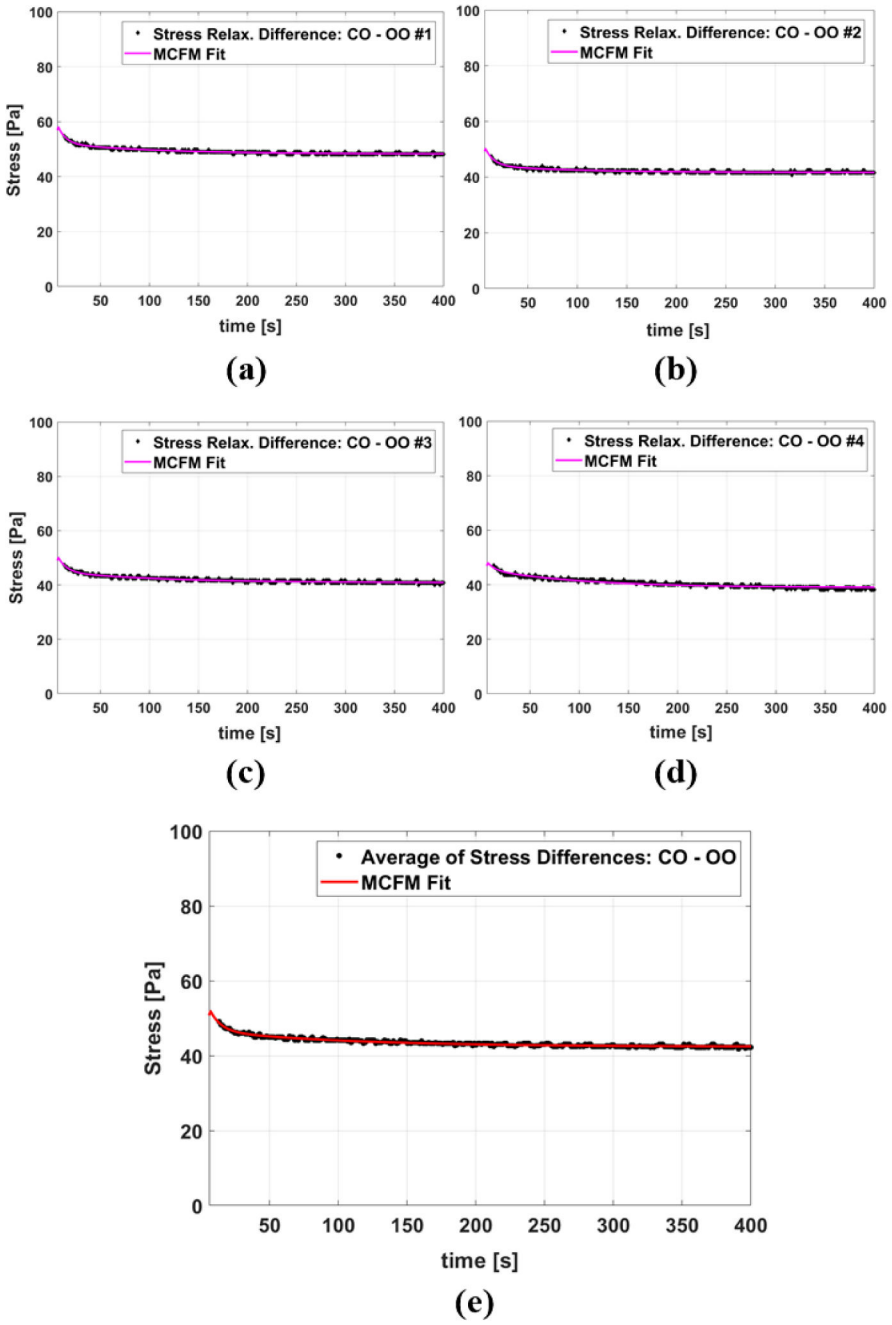
**(a)-(d)** MCFM fit to the stress relaxation differences between some individual castor oil CIP and olive oil CIP samples. (e) MCFM fit to the average of stress differences.

Figures 5 (a)-(d) show four examples of the MCFM curve fits to the stress differences of samples with castor oil CIPs and samples with olive oil CIPs, and Figure 5 (e) presents the fitting results when the averages of all stress differences of the two groups are taken into account. The results of fitting parameters are reported in Table 3 with the value of fitting goodness evaluated by R^2^. The fitting results for the two time constants show that the higher time constant (which is associated with the castor oil) is, on average, approximately 12 times larger than the smaller time constant (which is associated with the olive oil).

### 4.2 Perfused Placenta *in vitro* elastography

**Figure 6.**
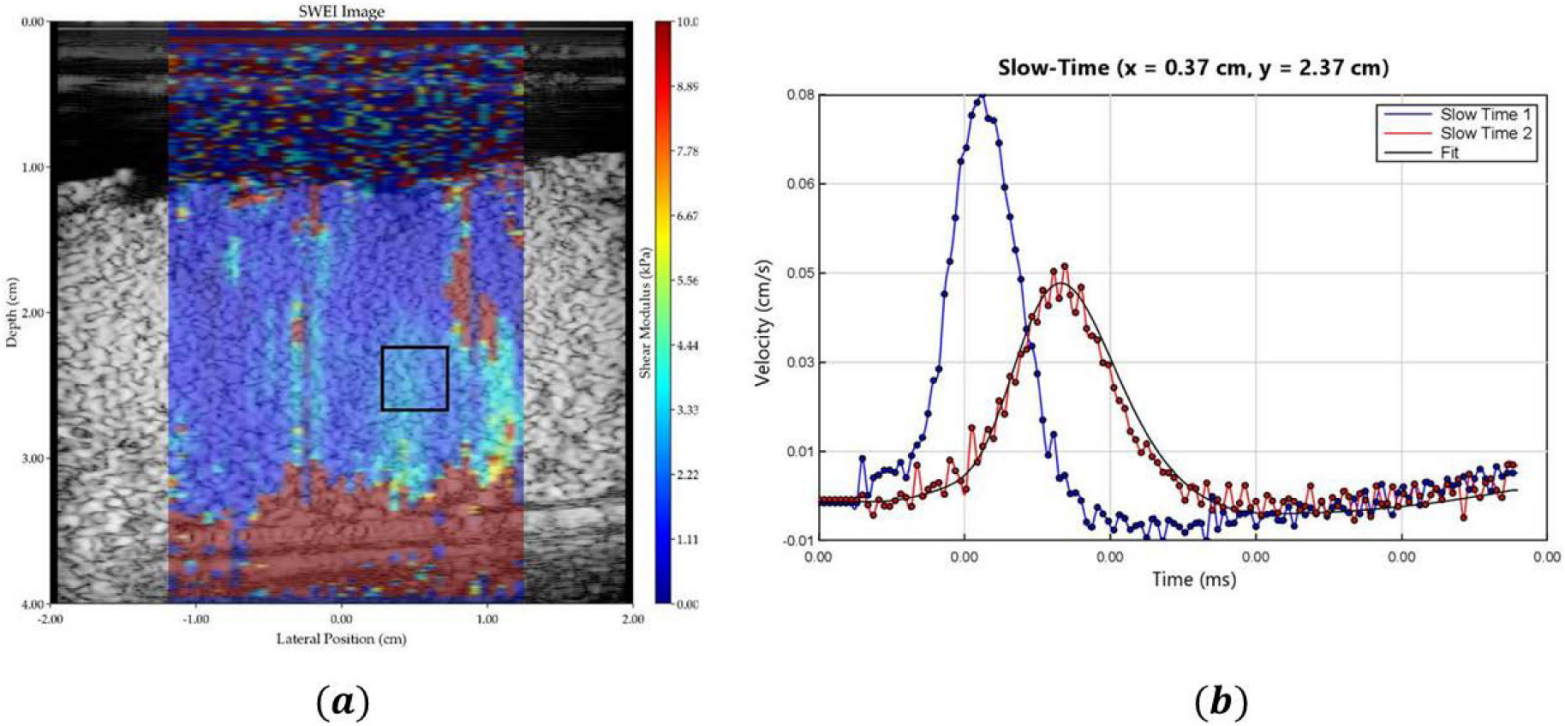
Elastography results from a perfused placenta. **(a)** B-scan image with tissue stiffness as a pseudo-color overlay. Blue represents softer and red indicates harder region. **(b)** Shear waves from radiation force push pulses at 2 locations. These data form the basis of elastography measurements.

Figure 6 (a) shows the elasticity measurements as a color image overlaid on the B-scan of the perfused placenta and Figure 6 (b) illustrates the shear wave generated in the tissue due to the application of acoustic radiation force push pulses at two locations that are used for elastography measurements.

**Figure 7.**
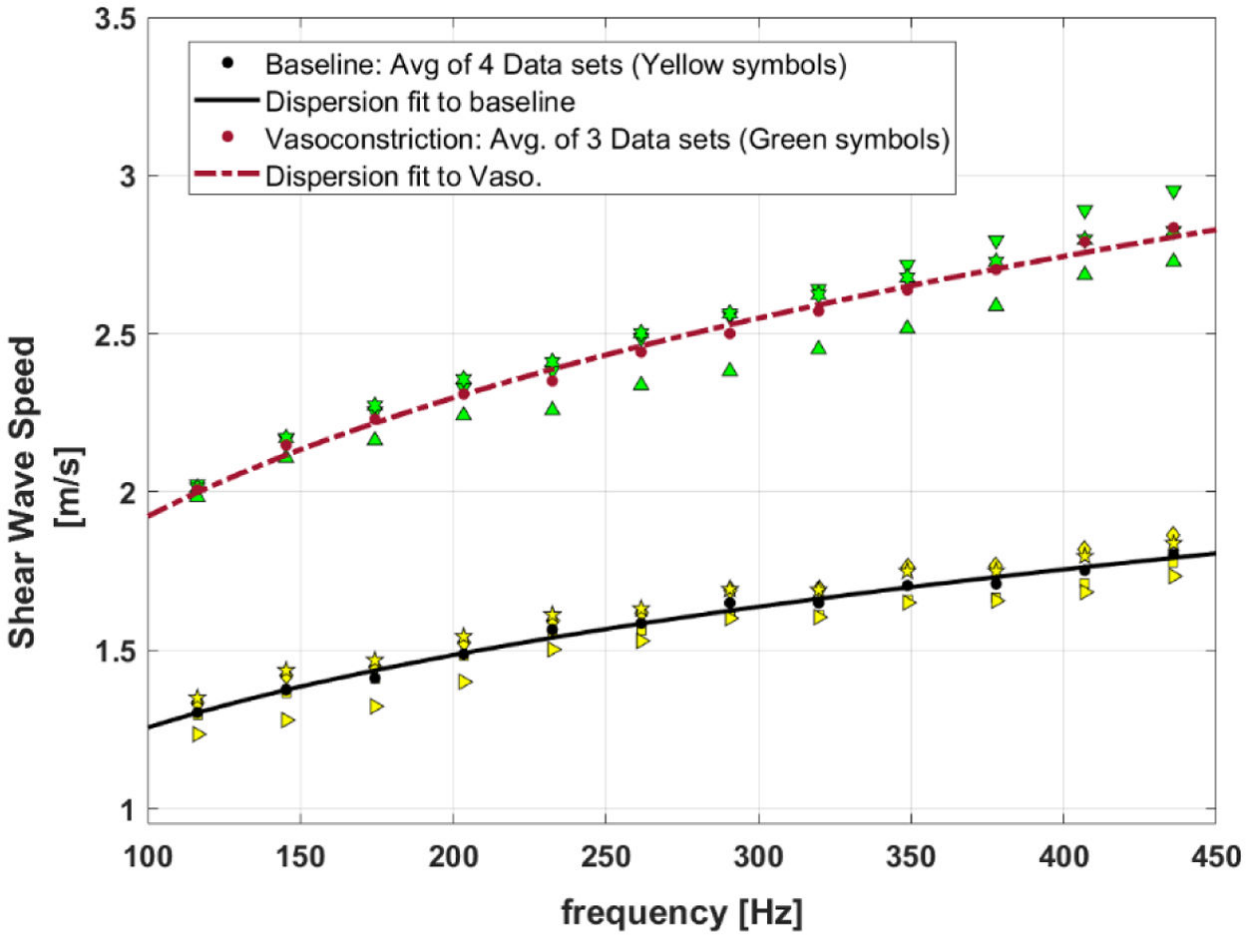
SWS dispersion curves of baseline placenta versus vasoconstriction condition. The fitted curves are results of power-law fitting to the average of measured data sets for each condition. Yellow symbols: 4 datasets of baseline measurements. Green symbols: three datasets of vasoconstriction measurements.

Figure 7 presents the SWS dispersion curves for baseline and the vasoconstriction condition for the perfused post-delivery placenta. Introducing vasoconstrictor agents causes a decrease in vessel diameters and, therefore, an increase in the SWS measurements. The higher level of SWS for the vasoconstriction implies that the tissue is stiffened in comparison to the baseline. In this figure, the SWS dispersion measurements are presented as four data sets for baseline, and three datasets for vasoconstriction, shown as different yellow symbols and green symbols, respectively.

The MCFM framework predicts a power law frequency-dependent elasticity *E*(*ω*) and SWS *c_s_* (*ω*) for the soft vascularized tissues as *E*(*ω*) ∼ *A* ∙ ∣ω∣^*a*^ and *c_s_* (*ω*) ∝ *ω*^*a*/2^ with *a* as the power law parameter related to the vasculature networks of the tissues (*a* = *b*−1) and *ω* = 2*π f* [30]. Therefore, the curve fit to the dispersion of SWS for baseline and vasoconstriction can approximate the key power law behavior at the two states. In Figure 7, the power law curve fits to the averaged SWS dispersion measurements for each condition are shown as lines, and the fitting coefficients are reported in Table 4. The results indicate that the power-law parameter *b* from the SWS dispersion measurement fitting is slightly higher in vasoconstriction in comparison to the baseline.

## 5. Discussion

Employing the MCFM offers the potential to investigate the role of the fractal vasculature of tissues and the fluid contents as they influence tissue stiffness at a macroscopic scale. According to the MCFM derivation, each vessel in a vasculature is associated with a characteristic time constant *τ_char_* and therefore a branching vasculature, comprised of different vessels from smallest to largest, introduces a spectrum of time constants that contribute to the behavior of the soft tissue under study. Under the MCFM, an increase in the time constant is reflected as increases in the stress relaxation level and the measured stiffness of the tissue. To investigate what could change the time constant itself in a tissue, the MCFM suggests that for a simple block of tissue with a vessel of fluid, factors such as vessel radius, fluid viscosity, and tissue stiffness would change the time constant according to *τ_char_* ∝ (*η*/*Er*^4^).

In gelatin-based phantom experiments, it is shown that when fluid within the CIP is changed from a low viscous oil (olive oil) to a fluid with higher viscosity (castor oil), the stress relaxation curves shift to higher values under the same strain applied in the test. Moreover, it is shown that the MCFM fits well to the stress differences of olive oil CIP samples and castor oil CIP samples, containing two fitted time constants where the larger one (which is associated with the castor oil) is approximately 12 times larger than the smaller time constant (which is associated with the olive oil) in the fitting process. This is close to the range predicted by the theory using the associated parameters as:

(16) $$\frac{\tau_{{}_{CO}}}{\tau_{{}_{OO}}}=\left(\frac{\eta_{{}_{CO}}}{\eta_{{}_{OO}}}\right)=12.25$$

where, by substituting our experimental parameters into Equation (5):

(17) $$\tau_{{}_{CO}}=\frac{0.98(0.035^{{}^{2}})(0.038)}{\frac{\pi }{8\times 0.032}(0.0015^{{}^{4}})(7000)}=104.5\;s$$

which is approximately close to the time constant estimated for castor oil from the stress relaxation curve-fitting reported in Table 3, and indicates the agreement of the MCFM prediction in theory and the experiment.

Next, considering the fractal vasculature within the placenta, the injection of the vasoconstriction agent imposes a decrease in the radii of vessels, which also results in an increase in arterial pressure. The elevated dispersion curve for the vasoconstriction condition in the placenta demonstrates that vasoconstriction imposes a stiffening effect on the tissue. In these experiments, the arterial pressure under a constant flow pump increased from approximately 40 mmHg to 90 mmHg after vasoconstriction. Under Poiseuille’s law, this corresponds to a decrease in radius to the fourth power using Equation (1), which is related to the vessel radii scale factor X according to Equation (8). The relationship of the arterial pressure change with the vessel radii scale factor can therefore be written as:

(18) $$\frac{\Delta P_{2}}{\Delta P_{1}}={\left(\frac{r_{1}}{r_{2}}\right)}^{4}=\frac{1}{\chi^{4}}$$

(19) $$\chi ={\left(\frac{40}{90}\right)}^{{}^{1∕4}}=0.8165≅81.7\%$$

We can roughly estimate how the stress behavior and therefore, the stiffness level of the placenta could change between baseline and the vasoconstriction state with the scale factor of X=0.817. Borrowing from the dispersion fitting results presented in Table 4, the power law parameter (*b* = *a* + 1) approches 1.5 for the vasoconstriction. Using Equation (11) for the stress response when *b* is equal to 1.5, the leading term in X simplifies, predicting a new stress response changed approximately by 1/*χ*^2^. This implies that the stress level increases and the tissue becomes stiffer due to vascoconstriction (X<1). In our case, the prediction from theory is that the post-vasoconstriction stress relaxation would be elevated by a factor of (1/0.817)^2^ which is approximately 1.5, very close to the proportional change shown in Figure 7.

Specifically, by examining Figure 7 and the fitting results in Table 4, the second fitted parameter *c′* from the SWS dispersion fitting for the baseline and vasoconstriction is 0.412 and 0.589, respectively. The proportion of these two parameters is 0.589/0.412 = 1.4 which indicates a higher level of ***c′*** for vasoconstriction, consistent with the earlier estimate following Equations (18) and (19).

Thus, the leading term X defined in the MCFM derivations in Equations (7)-(11) appears to adequately predict observed changes in tissue stiffness caused by vasoconstriction.

## 6. Conclusions

By employing Poiseuille’s law of fluid flow, the MCFM introduces a characteristic time constant associated with each vessel in a vasculature that is found to affect the stress-strain behavior in the underlying vascularized soft tissue. In this study, by using two separate experiments in gelatin-based phantoms and also perfused post-delivery placenta, the effect of changing this time constant on the tissue behavior is investigated. Under this framework, we examined the changes in two separate parameters:

- vessel fluid viscosity (in phantom experiments)
- vessel diameter (through vasoconstriction in placenta).

The associated time constants were changed, and it is shown in both cases that increasing the characteristic time constants creates a stiffening effect on the tissue/phantom under study through increased stress relaxation level in phantoms and elevated SWS dispersion in placenta. The degree of change is also consistent with the prediction of MCFM. Thus, the MCFM can be useful for stress-strain models of vascularized tissues including changes over time as pathologies or vasoactive responses are activated.

## Figures and Tables

**Table 1.** Portion of ingredients used for making phantoms.

Ingredient	Amount
degassed water	900 mL
gelatin	4%
NaCl	0.9%
agar	0.15%

**Table 2.** Mean viscosity measurements of the selected oils and their standard deviations (SD) at room temperature.

Oil	Dynamic viscosity (Pa·s)	SD
castor oil	0.98	0.04
olive oil	0.08	0.001

**Table 3.** MCFM fitting results.

Fitting case		*B_CO_*	*τ_CO_*	*B_OO_*	*τ_OO_*	*δ*	*R* ^2^
Difference between CO and OO samples	(a)	0.57	102	1.29	8.03	48.19	0.912
	(b)	0.36	95	1.28	8.5	41.67	0.85
	(c)	0.694	105	1.45	8.64	40.85	0.91
	(d)	0.958	98	0.59	7.8	38.96	0.913
Average of stress differences	(e)	0.61	105	1.16	8.1	42.37	0.949

**Table 4.** Power law fitting to dispersion of SWS: *c_s_* = *c′* · *f^a′^* (*a′* = *a*/2).

Condition	*c′*	*a′*	*R* ^2^
baseline	0.412	0.242	0.99
vasoconstriction	0.589	0.257	0.9915
